# The Role of Oxidative Stress in Heart Failure: Mechanisms and Therapeutic Targets

**DOI:** 10.7759/cureus.111384

**Published:** 2026-06-23

**Authors:** Taha Hussain, Asmi Pandey, Dipesh Karki, Prashant Neupane

**Affiliations:** 1 Faculty of Biology, Medicine and Health, University of Manchester, Manchester, GBR; 2 Department of Internal Medicine, Royal Liverpool University Hospital, Liverpool, GBR; 3 Department of Internal Medicine, Manchester University NHS Foundation Trust, Manchester, GBR; 4 Department of Vascular Surgery, Manchester Royal Infirmary, Manchester, GBR

**Keywords:** antioxidant capacity, heart failure, mitochondrial calcium, mitochondrial-targeted therapy, nad/nadh metabolism, reactive oxygen species (ros)

## Abstract

Heart failure is characterised by increased production of reactive oxygen species (ROS), which contribute to apoptotic signalling. Changes in excitation-contraction coupling in heart failure promote increased calcium extrusion and decreased calcium uptake by myocardial mitochondria. Mitochondrial calcium is required to increase the Krebs cycle flux to generate sufficient nicotinamide adenine dinucleotide (NADH) to match the increased energy demand seen in heart failure. Reduced NADH availability can decrease levels of reduced nicotinamide adenine dinucleotide phosphate (NADPH), leading to a loss of NADPH-mediated antioxidant capacity and thereby increasing ROS emission and oxidative damage. Accordingly, a group of therapies that increase mitochondrial calcium, such as sodium glucose-linked transporter 2 inhibitors and ranolazine, have been investigated as potential treatments in heart failure. Proposed mechanisms include reducing calcium extrusion from mitochondria via the Na^+^/Ca^2+^/Li^+^ exchanger. A second group of emerging therapies targets oxidative stress by directly scavenging ROS (MitoQ and SS-31) and has also been studied for use in heart failure. Contemporary research developments propose an alternative approach to targeting oxidative stress by enhancing endogenous antioxidant capacity through the administration of nicotinamide adenine dinucleotide (NAD) precursors, thereby increasing intracellular NAD availability.

## Introduction and background

Heart failure is a clinical syndrome in which the heart is unable to pump sufficient blood around the body to meet metabolic demands. This leads to a range of symptoms that can significantly impair functional capacity and quality of life. For example, pulmonary oedema secondary to heart failure can present as exertional dyspnoea, paroxysmal nocturnal dyspnoea and orthopnoea [1]. Heart failure is a major cause of worldwide mortality and morbidity, particularly in ageing populations and individuals with obesity, due to a higher prevalence of heart failure in these groups [2-4].

Although heart failure is a multifactorial disease with diverse aetiologies, oxidative stress has emerged as a promising therapeutic target. Reactive oxygen species (ROS) are highly reactive chemical species formed from oxygen molecules as part of normal cellular physiology. At low intracellular concentrations, ROS play an essential role in cellular signalling cascades. However, at higher concentrations, they become pathological and can cause extensive damage to various cellular components, including peroxidation of lipids and oxidative damage to DNA and proteins, ultimately leading to apoptosis. Therefore, cells have a variety of mechanisms to protect them from ROS-induced oxidative damage. Oxidative stress or redox imbalance refers to a mismatch between ROS production and detoxification via cellular defences [5].

Oxidative stress is a key feature in the progression of heart failure, as demonstrated in both clinical and in vitro studies [6-8]. This review will primarily focus on four key areas: mechanisms of ROS production and detoxification; the role of dysfunctional calcium handling in failing cardiomyocytes as a significant contributor to ROS production; therapies that target cardiac oxidative stress by either targeting calcium handling or by directly targeting ROS; and the future potential of targeting oxidative stress by enhancing endogenous antioxidant capacity. 

## Review

Physiology of reactive oxygen specie*s*

ROS are produced in cells from many different sources as part of normal physiological processes. Although mitochondria are the primary site of ROS production, additional sites will be discussed first [9]. NADPH oxidase (NOX) is a membrane-bound multi-subunit enzyme whose function is to reduce oxygen (O_2_) to superoxide (O_2_^-^) using nicotinamide adenine dinucleotide phosphate (NADPH) as an electron donor; enzymes can use NADPH to transfer electron(s) to molecules to reduce them [10]. NOX is activated upon binding of GTP to the Rac1 subunit. Similarly, lipidation of Rac1 can also lead to NOX activation. Interestingly, HMG-CoA reductase inhibitors (statins) prevent lipidation of Rac1 and decrease NOX-mediated ROS emission. This antioxidant effect may contribute to the efficacy of statins in reducing cardiovascular mortality, although its clinical significance remains uncertain [11].

Xanthine oxidase (XO) is an intracellular enzyme that also generates ROS. XO is responsible for oxidising hypoxanthine to xanthine, which is further oxidised to uric acid, to be excreted by the kidneys. XO carries out these oxidation reactions by using O_2_ as an electron acceptor and therefore reduces it to produce O_2_^-^ (incomplete reduction) and H_2_O (complete reduction) [12]. 

Uncoupled nitric oxide synthase (NOS) is a source of ROS in heart disease; NOS is a dimeric enzyme whose function is to produce nitric oxide [13]. A cofactor of NOS, called BH_4_, becomes oxidised in the presence of ROS during cardiac injury, which causes NOS uncoupling. This structural change induces the uncoupled NOS to produce O_2_^-^ instead of nitric oxide, thus driving oxidative stress; this is a significant source of ROS in heart failure, and potential therapies have been trialled to reduce uncoupled NOS-mediated ROS emission [13]. 

The largest source of ROS production in cells is due to the movement of electrons through the electron transport chain (ETC) during oxidative phosphorylation. During this process, reduced co-enzymes NADH and FADH_2_ are oxidised to transfer their electrons to the ETC. As these electrons move through complexes I to IV of the ETC, the energy released is utilised by the complexes to generate a proton gradient across the inner mitochondrial membrane, which is then used by complex V to generate ATP via ATP synthase activity [14]. Furthermore, at complex IV, the electrons are transferred to O_2_, reducing it to water, which is then exhaled. However, during this process, some electrons may leave the ETC before reaching complex IV and interact with O_2_, thereby incompletely reducing it to form O_2_^-^ [14,15]. This production of ROS is inevitable as cells carry out metabolism; therefore, cells have evolved mechanisms to detoxify ROS. During heart failure, there is a mismatch between myocardial ATP demand (which requires oxidative phosphorylation) and supply, which leads to enhanced ROS emission, thereby contributing to oxidative cardiac damage [16].

To counter the damaging production of ROS, the body employs specific mechanisms [17]. Superoxide dismutase (SOD) is an enzyme that converts O_2_^-^ to H_2_O_2_, which is then reduced by glutathione peroxidase (GPx) to form water and O_2_. GPx acts by using two glutathione (GSH) molecules to reduce H_2_O_2_, thus yielding two molecules of oxidised GSH in the process. Therefore, for GPx to continue its action, it requires a pool of reduced GSH. This pool is maintained by another enzyme called glutathione reductase, which uses its cofactor NADPH to reduce the oxidised GSH, allowing it to be used by GPx again. This cycle is the key mechanism by which the body maintains a normal redox state [18]. Non-enzymatic antioxidants, such as vitamin E and C, also contribute to cellular defences against ROS by various mechanisms. For example, vitamin C may prevent NOS uncoupling [19]. A balance between ROS production and antioxidant capacity is critical to cellular redox homeostasis, as excessive ROS production can induce cellular damage via a variety of mechanisms, including lipid peroxidation and protein oxidation [20,21]. Furthermore, excessive mitochondrial ROS production can lead to the opening of mitochondrial permeability transition pore (mPTP) channels in the mitochondrial membrane, causing membrane instability, which allows the transfer of mitochondrial apoptotic factors into the cytosol [22,23].

In heart failure, antioxidant defences such as SOD, GSH and catalase are reduced, whereas pro-oxidant pathways such as NOS, NOX, XO, and oxidative phosphorylation are enhanced [24]. This results in a net increase in ROS, subsequently leading to an elevated ratio of oxidised GSH to reduced GSH, which is commonly seen in patients with heart failure and is a marker of poor redox status [25]. In addition, nicotinamide adenine nucleotide (NAD) is an integral part of the antioxidant defence system as it is a precursor to NADPH, which provides the reducing power to maintain physiological levels of reduced GSH [26]. Therefore, decreased NAD levels may contribute to oxidative stress; studies have found that patients with heart failure have reduced expression of an enzyme called nicotinamide mononucleotide adenyl transferase, which is involved in the synthesis of NAD [26].

Mitochondrial calcium physiology

Mitochondria have an intermembrane space between the outer and inner membranes. The matrix is the largest mitochondrial compartment, which lies beneath the inner membrane [27]. The outer membrane contains an abundance of voltage-dependent ion channels, which allow calcium (Ca^2+^) to move into the intermembrane space. Calcium then moves through the inner mitochondrial membrane and into the matrix via the mitochondrial calcium uniporter (MCU), which allows Ca^2+^ to move down its electrical gradient. The MCU is a complex made up of several proteins that serve as ion channels for Ca^2+^. This is the primary mechanism by which calcium can enter the mitochondria [28,29].

Mitochondrial Na^+^/Ca^2+^/Li^+^ exchanger (NCLX) is the main way in which calcium is extruded from mitochondria to maintain normal mitochondrial calcium levels in response to changes in cytosolic calcium. NCLX is found on the inner mitochondrial membrane and works via an antiporter mechanism; it utilises the movement of sodium into the mitochondria down its concentration gradient to export calcium in the opposite direction [30,31]. The kinetics of this protein are slower than the mechanism of calcium influx, which is essential for allowing mitochondrial calcium accumulation during beta stimulation. This process is vital to match energy demand and supply, as calcium stimulates the Krebs cycle and, consequently, ATP generation [32-34]. However, in heart failure, elevated cytosolic Na^+^ may favour NCLX-mediated calcium extrusion from mitochondria instead, thereby limiting calcium accumulation [30]. In addition, the sodium proton exchanger (NHE) is a regulator of NCLX-mediated calcium extrusion from mitochondria. This is because it pumps Na^+^ from mitochondria into the cytosol while pumping protons into mitochondria, maintaining a Na^+^ gradient across the mitochondrial membrane, which NCLX then uses to extrude calcium [27].

There are close connections between the mitochondria and the sarcoplasmic reticulum (SR), known as mitochondria-associated endoplasmic reticulum membranes, which allow calcium to move directly from the SR into the mitochondria, thus maintaining mitochondrial calcium levels [35,36]. This mechanism also allows calcium levels to increase in mitochondria during periods of high energy demand, stimulating the Krebs cycle (by activating dehydrogenase enzymes) to regenerate NADH and FADH_2_, which can then donate electrons at the ETC to increase ATP production [37]. Mitochondrial-associated endoplasmic reticulum membranes consist of various proteins that form complexes facilitating the transport of Ca^2+^ from the SR into mitochondria, thus serving as another important mechanism of mitochondrial calcium regulation [36].

Dysfunctional mitochondrial calcium handling in failing cardiomyocytes

Under physiological conditions, when the sarcolemma is depolarised due to an action potential, it causes the SR to release calcium via RyR2 channels, resulting in a cytosolic calcium spike, which causes systole. For diastolic relaxation to occur, the cytosolic calcium levels must be returned to baseline; this occurs via the action of sarco/endoplasmic reticulum calcium ATPase (SERCA), which transports calcium back into the SR, and the NCX (found on the sarcolemma), which exports calcium out into the extracellular space [38]. This process of excitation-contraction coupling is altered in heart failure, leading to pathological intracellular concentrations of sodium and calcium, ultimately resulting in oxidative stress [8].

In heart failure, the cytoplasmic concentration of sodium is increased, and the systolic Ca^2+^ concentration is decreased due to a reduced Ca^2+^ content of the SR [39]. These changes in ion concentrations hinder mitochondrial Ca2+ uptake. Furthermore, connections between the SR and mitochondria, called mitochondrial-associated membranes, mean that decreased Ca^2+^ release from the SR will result in decreased Ca^2+^ uptake by mitochondria [37,40]. In addition, the elevated Na^+^ concentration in failing cardiomyocytes accelerates Ca^2+^ removal from mitochondria by increasing NCLX activity [41]. The disruption of mitochondrial-associated membranes also hampers mitochondrial calcium uptake. This is partly due to downregulation of SR-mitochondrial tethering proteins such as mfn2 [42,43].

Changes in excitation-contraction coupling seen in heart failure may lead to increased calcium extrusion and decreased calcium uptake by the cardiac mitochondria. Reduced mitochondrial calcium impairs Krebs cycle flux, which limits NADH regeneration and thereby contributes to enhanced mitochondrial ROS emission [44].

The decrease in mitochondrial Ca^2+^ levels also causes an energy-demand mismatch. During beta stimulation, enhanced mitochondrial Ca^2+^ uptake increases Krebs cycle flux by activating pyruvate dehydrogenases, which are the key enzymes in this cycle [45]. Krebs cycle activity generates a substantial amount of NADH, which can become oxidised at the ETC to generate ATP. In this manner, low mitochondrial Ca^2+^ levels hinder oxidative phosphorylation because NADH cannot be generated and oxidised at the electron transport chain, which decreases ATP synthesis and ultimately results in energy failure [46]. Experimentally, it was shown that NADH oxidation at the ETC was increased after enhancing mitochondrial Ca^2+^ levels, which highlights the critical role of calcium in matching energy demand and supply in cardiomyocytes [47].

An increase in Ca^2+^-mediated oxidative phosphorylation would result in increased ROS emission at the ETC. To counteract this, another significant role of Ca^2+^ is to improve the antioxidant capacity of cells during times of high demand by increasing NADH production (as discussed above), which leads to increased NADPH production because NADH can act as a precursor to NADPH [48,49]. 

In summary, decreased mitochondrial calcium levels can lead to decreased NADH levels, which in turn lead to decreased NADPH levels, which negatively affect mitochondrial antioxidative capacity because NADPH is needed to maintain a reduced pool of many redox couples, such as GSH. In conclusion, low mitochondrial calcium leads to low NADH and NADPH, which causes energy supply demand mismatch as well as decreased antioxidant capacity [44,50].

The enzyme mitochondrial transhydrogenase catalyses a reversible reaction linking NADH and NADPH, known as the nicotinamide nucleotide transhydrogenase reaction. The reaction taking place under physiological conditions is NADH + NADP^+^ <=> NADPH + NAD^+^. This is an essential reaction as NADPH is responsible for maintaining redox homeostasis [18]. However, when there is a pathological increase in cardiac workload (as seen in heart failure), the reaction is reversed to favour NADH production to generate ATP at the expense of NADPH-mediated antioxidative capacity. Furthermore, under pathological workload, when there is not a sufficient increase in mitochondrial Ca^2+^ levels to match energy demand, NADH oxidation to produce ATP at the ETC is greater than the Ca^2+^-mediated increase in Krebs cycle activity to regenerate the NADH. This leads to a build-up of NAD, which further favours the reverse reaction to maintain equilibrium, ultimately contributing to increased ROS emission [18,51].

ROS derived from multiple sources can activate several redox-sensitive channels in the inner mitochondrial membrane, including the permeability transition pore (PTP), inner mitochondrial membrane anion channel (IMAC) and ATP-sensitive K^+^ channel [52-54]. This results in the movement of ions across the membrane, which causes the mitochondrial membrane potential to become disrupted [55]. As a result, ETC activity increases in an attempt to maintain membrane potential. However, ETC activity requires oxidation of NADPH and NADH to provide electrons for the ETC, which consumes the intracellular pool of NADPH and NADH [55].

Furthermore, as discussed previously, increased ETC activity also leads to increased ROS emission, which ultimately negatively affects the antioxidative capacity of the cell. ROS can leave mitochondria via mPTP and IMAC channels to trigger ROS release from neighbouring mitochondria, resulting in more ROS-mediated damage; this concept is known as ROS-induced ROS release [55]. Mitochondrial apoptotic factors (e.g., cytochrome C) can also leak out via these mPTP channels [56]. In conclusion, in heart failure, a decrease in mitochondrial calcium concentration causes an energy deficit as well as increased ROS emission, which then causes damage to neighbouring cardiomyocytes, thereby establishing a vicious cycle.

Therapeutic targets

Ranolazine

Ranolazine is well established as an anti-anginal drug; however, it also has effects on ROS emission by inhibiting the late sodium current [57]. The late sodium current describes the influx of sodium into the cardiomyocytes during the repolarisation phase of the cardiac action potential [57]. Under physiological conditions, this current is minimal; however, it is often significantly elevated in heart disease, resulting in the high cytoplasmic sodium concentrations often seen in failing cardiomyocytes. This elevated cytoplasmic sodium concentration will result in enhanced activity of the sarcolemma sodium-calcium exchanger protein, which will drive calcium into the cell in exchange for extruding sodium into the extracellular space [58,59]. Therefore, treatment with ranolazine may inhibit calcium influx into the cellular cytoplasm by inhibiting this late sodium current, which may prevent oxidative damage secondary to cytoplasmic calcium overload [60,61]. This is evidenced by studies that show that blocking NCX activity reduces the rise of intracellular calcium during ischemia-reperfusion injury [62]. To reinforce this effect, it has been shown that inhibiting the late sodium current using sodium channel blockers leads to significantly reduced intracellular calcium levels [63].

Ranolazine may also reduce the intracellular cytoplasmic sodium concentration by inhibiting the late sodium current. This could reduce NCLX-mediated calcium extrusion from the mitochondria, thereby enhancing mitochondrial antioxidant capacity, although this mechanism of ranolazine remains poorly characterised in the literature [51]. However, evidence from preclinical studies shows that the late sodium current observed in heart failure depletes mitochondrial calcium levels by enhancing mitochondrial calcium efflux via NCLX, which in turn leads to increased ROS emission by reducing the cell's antioxidant capacity [64]. Therefore, evidence suggests that blocking the late sodium current decreases mitochondrial ROS emission and may be a therapeutic target to treat heart failure [65].

As discussed previously, scientific literature and preclinical evidence suggest that increasing mitochondrial calcium accumulation by inhibiting late sodium current reduces ROS emission. However, there are few human clinical trials to determine whether this effect of ranolazine translates into clinical efficacy [66]. There have been preclinical studies examining the antioxidant effects of ranolazine in animal models at doses similar to those used in clinical trials for its other uses. These studies found that ranolazine preserved ATP levels and ventricular contractility during episodes of ischemia. Necrosis was also found to be decreased using biomarkers of necrotic damage [67]. Several other preclinical studies have reported the beneficial effects of ranolazine across multiple causes of cardiac dysfunction [68-70]. However, a clinical trial studying the effects of ranolazine on diastolic heart failure with preserved ejection fraction did not demonstrate improvement in left ventricular relaxation [71].

Cardiomyocytes from patients with this type of heart failure still show a high pathological calcium overload under conditions of stress, despite treatment with ranolazine. However, this is not thought to be mediated by the NCX exchanger or related to sodium movements across the membrane, which may explain the lack of efficacy [72]. Furthermore, another randomised controlled trial in 2018 found that ranolazine did not show any efficacy in patients with non-obstructive hypertrophic cardiomyopathy (a variant of diastolic heart failure) [73]. 

Heart failure with preserved ejection fraction remains a significant clinical challenge due to the limited availability of established targeted therapies. Therefore, more research is warranted to understand the molecular pathophysiology of diastolic heart failure and to determine the potential therapeutic role of ranolazine in this context.

SGLT2 Inhibitors 

Sodium glucose-linked transporter 2 (SGLT2) inhibitors are a relatively recent class of drugs initially developed to treat type 2 diabetes mellitus. A clinical trial found that empagliflozin (an SGLT2 inhibitor) reduced heart failure mortality in diabetic patients [74]. This result is thought to be linked to several effects exerted by these drugs. For example, by increasing urine excretion by the kidneys, SGLT2 inhibitors lead to a decrease in the circulating fluid volume, which in turn leads to a favourable reduction in blood pressure [75]. Interestingly, recent data suggest many of the SGLT2 inhibitors also inhibit the sarcolemma Na^+^/H^+^ exchanger (NHE) by binding to the Na^+^ binding site on the exchanger, which favours Ca^2+^ accumulation in cardiac mitochondria [76]. However, it is not known to what extent, if any, this effect of SGLT2 inhibitors contributes to the reduction in mortality seen in patients with heart failure; this effect is being actively investigated, and several clinical trials are in progress [77,78].

MitoQ

MitoQ consists of two components: a ubiquinone group (active moiety) and a triphenylphosphine lipophilic cation. The positive cation allows it to move into the mitochondria and accumulate 100-fold in the inner mitochondrial membrane [79,80]. This is because the mitochondrial membrane has a highly negative membrane potential due to the chemical gradient of protons across the inner mitochondrial membrane, which is necessary to synthesise ATP by the electron transport chain. In the inner mitochondrial membrane, MitoQ is reduced to its active form, ubiquinol, by complex 2 of the respiratory chain. Ubiquinol can become oxidised to ubiquinone by ROS (which are in turn reduced) as it scavenges them. Ubiquinone can then be reduced back to ubiquinol by electrons from complex 2, meaning it can be reused repeatedly to scavenge ROS due to its regenerative capacity [81].

Several studies have evaluated the therapeutic effects of MitoQ in various models of cardiac pathology, including ischemia-reperfusion injury and hypertension [82,83]. However, there are minimal studies that have investigated its effects in heart failure specifically. One study examined the impact of MitoQ on the development of pressure overload-induced heart failure in rats 14 days post-treatment [84]. The study found that MitoQ did not reduce left ventricular hypertrophy; however, it did reduce right ventricular hypertrophy and pressure-induced fluid congestion in the right lung. Furthermore, the study found that heart failure resulted in a loss of mitochondrial function, which was reversed by MitoQ treatment, greatly enhancing mitochondrial function and tissue respiratory capacity. The study found the primary mechanism by which MitoQ improved mitochondrial function was by decreasing mPTP opening and decreasing hydrogen peroxide formation [85].

However, there are two key issues raised with the use of MitoQ in heart failure. Firstly, in heart failure, the cardiac mitochondria are in a highly oxidised state (due to the production of excess ROS), which disrupts the activity of the respiratory chain, which may hinder regeneration of ubiquinol and therefore promote accumulation of ubiquinone, which has been shown to exert pro-oxidative effects at high concentrations [84]. Accordingly, more studies are required to explore the effects of MitoQ specifically in the context of heart failure. 

SS-31

Cardiolipin is a phospholipid in the inner mitochondrial membrane and is vital for the proper function of the electron transport chain. It maintains the structural architecture of the mitochondrial cristae and therefore keeps the integrity of the mitochondrial electron transport chain [86]. Cardiolipin interacts with cytochrome c to form a complex (cardiolipin/cytochrome c complex), which allows efficient transfer of electrons from complex III to IV of the electron transport chain. However, in the presence of H_2_O_2_, cardiolipin undergoes hydrophilic binding to cytochrome c, which causes cytochrome c to act as a peroxidase, resulting in cytochrome c-mediated oxidation of cellular components. 

This causes extensive damage to the mitochondrial membranes as well as the electron transport chain. Ultimately, this can lead to cytochrome c “leaking” out into the cytoplasm, causing apoptosis [86]. SS31 binds to cardiolipin and disrupts the interaction between cardiolipin and cytochrome c, which pushes cytochrome c towards its electron carrier function [87]. Therefore, the mechanism of SS31 is to decrease the peroxidase activity of cytochrome c and promote its normal function, which is to transfer electrons between respiratory chain complexes [88]. The chemical structure of SS31 allows it to accumulate in the inner mitochondrial membrane independent of the mitochondrial membrane potential, which means it can also target diseased mitochondria. This is unlike MitoQ, which requires a conjugated cation to utilise the membrane potential to move into mitochondria.

A 2017 double-blinded randomised controlled trial investigating the efficacy of elamipretide (SS-31) on 36 patients with heart failure with reduced ejection fraction reported notable findings. It concluded that a single infusion of elamipretide was well-tolerated and suggested a clear dose-response relationship with higher doses associated with a favourable decrease in left ventricular end systolic and diastolic volume. This is the first human study of SS-31. The small cohort size warrants more research to investigate the long-term effects and tolerability of SS-31 in humans [89].

In a 2016 study, 14 dogs with heart failure secondary to micro-emboli were randomised to three months of treatment with either SS-31 or saline. Sabbah et al. showed that chronic therapy with SS-31 (elamipretide) improved left ventricular systolic function and myocardial mitochondrial function in dogs with advanced heart failure [90]. Though trials of SS-31 on human heart failure are limited, there are several in vitro studies. These preclinical studies have shown SS-31 to reduce ROS emission, mitochondrial damage and enhance cardiac function in the context of heart failure and ischemic damage [91,92].

Transverse aortic constriction is a commonly used experimental model of heart failure. It involves severe pressure overload on the heart that leads to ventricular hypertrophy and subsequent heart failure. In this condition, a study found that SS-31 improved cardiac function by reducing cardiac hypertrophy and scarring, as well as enhancing mitochondrial bioenergetics [93]. Collectively, these findings suggest a mechanistic link between mitochondrial oxidative dysfunction and heart failure, although human evidence remains limited. Targeting mitochondrial ROS emission by SS-31 may translate into improved outcomes in clinical practice; however, there is not enough human trial data to confirm this. In conclusion, although studies show promising effects of SS-31, more research is warranted. 

NAD-based therapies

Antioxidant Role of NAD 

Enhancing endogenous antioxidant capacity may present as another strategy for targeting oxidative stress in heart disease. One way this can be achieved is by increasing NAD^+^ levels. During cardiac failure, NAD levels are low. NAD has many beneficial effects for cells, including the maintenance of redox homeostasis [94]. Importantly, NAD can act as a precursor to NADH, which acts as an electron donor to many ROS scavengers, such as GSH. This maintains a pool of reduced GSH, thereby contributing to a high ratio of reduced GSH to oxidised GSH, a key determinant of redox balance [94]. NAD is also known for exerting anti-inflammatory effects, which may also contribute to its therapeutic potential in heart failure [95,96]. In addition, NAD can exert antioxidant effects by activating several sirtuins (SIRTs), which are NAD-dependent protein acetylases that can modulate the antioxidant response. SIRTs are critical regulators of the antioxidant defence system by acting on many key targets [97,98]. One of these targets is NRF2, a master regulator of the antioxidant system that acts as a transcription factor for many antioxidant genes. De-acetylation of NRF2 by SIRT2 prevents ubiquitination of NRF2 by its key regulator, Keap1, which prevents degradation of NRF2, thus allowing it to translocate to the nucleus and bind to the antioxidant response element of genes to exert antioxidant effects [99]. Lastly, NAD is also critical for oxidative phosphorylation and ATP generation as it acts as a carrier to transport electrons to the ETC in the form of reduced NADH and FADH_2_ generated by the Krebs cycle [100].

Biosynthesis of NAD 

NAD can be synthesised via two main pathways. Most NAD is synthesised via the salvage pathway, in which nicotinamide (NAM) is converted to nicotinamide mononucleotide (NMN) by nicotinamide phosphoribosyl transferase (NAMPT); NMN can also be generated by phosphorylation of nicotinamide riboside (NR) by nicotinamide riboside kinase (NRK). These two pathways lead to the production of NMN, which is then converted to NAD by nicotinamide mononucleotide adenyl transferase (NMNAT) [101-103]. In heart disease, NAMPT is downregulated, whereas NRK is upregulated. The decreased NAMPT activity is thought to be a key reason for decreased cellular NAD levels during disease. This is because decreased NAMPT may lead to decreased NR, which is the substrate for NRK. As a result, NRK is unable to synthesise more NAD in the absence of exogenous NR because there is less substrate for NRK to act on [104]. This suggests NR supplementation could present as a viable strategy to increase NAD levels and, therefore, be proposed as a potential therapeutic target. The most widely used NAD precursor in studies is NR; one study reported that NR supplementation in murine models for cardiomyopathy may limit cardiac damage and preserve function in a NAD-dependent manner [104].

Clinical Use of NAD Precursors

Given the beneficial effects of NAD on cellular function, there have been many studies to determine whether replenishing NAD levels can serve as a potential therapeutic target for improving cardiovascular outcomes. Many of these studies involve the administration of the NAD precursor NR. A study found that NAD supplementation reduced cardiac fibrosis, necrosis, and inflammation in a mouse model with cardiomyopathy [105]. These effects of NAD supplementation are thought to be mediated by SIRT1 [105]. A study also found that NAD-dependent activation of SIRT1 and manganese superoxide dismutase in mice receiving NMN supplementation resulted in decreased oxidative stress, leading to a decline in age-related arteriole dysfunction [106]. Although these findings are promising, clinical evidence in patients with heart failure remains limited. Furthermore, there is a lot of variation in the results of existing studies. Whilst some studies show the beneficial effects described earlier, many studies do not report such outcomes. 

The NAD precursor used in many studies is nicotinic acid (NA). However, NA has been described for its lipid-lowering properties by acting on the GPR109A receptor; therefore, it is not certain whether the beneficial cardiovascular effects from NA supplementation are in fact due to increased NAD levels and subsequent SIRT1 activity [107,108]; almost all relevant studies conducted with NA describe improvements in lipid profiles [109,110]. However, it has also been proposed that SIRT1 may mediate this lipid-lowering activity because the concentration of NA needed to exert these clinically evident lipid-lowering effects is far higher than the concentration required for sufficient activation of the GRP109A receptor [111-113]. In addition, in some studies, NAD levels were not reported after administration of NAD precursors, which makes it difficult to determine whether drugs are acting via NAD-dependent mechanisms or not [109,114]. Furthermore, NAD is also known to cause uncomfortable symptoms for patients, such as pruritus, which raises further questions about its tolerability as a therapeutic agent in cardiovascular disease [115].

## Conclusions

Altered excitation-contraction coupling in heart failure results in impaired Ca2+ uptake and enhanced Ca2+ extrusion by cardiac mitochondria. This reduces Krebs cycle flux, thereby decreasing NADH production. This has two principal effects. Firstly, it creates an energy deficit, which contributes to ROS-induced cellular damage, and secondly, it reduces the endogenous antioxidant capacity of the cardiomyocyte. There are two major classes of therapies that target mitochondrial ROS emission. The first class of medications decreases intracellular sodium, thereby enhancing mitochondrial calcium accumulation (SGLT2 inhibitors and ranolazine). The second class of drugs directly scavenge mitochondrial ROS and prevent them from inducing cellular damage (MitoQ and SS-31). These drugs have shown promising results in preclinical studies. Notably, SGLT2 inhibitors have demonstrated improved cardiovascular outcomes in clinical trials. However, more studies are required to assess clinical outcomes and address significant gaps in the evidence base before most of these agents can be routinely adopted into standard care. Another promising strategy for targeting oxidative stress is to enhance endogenous antioxidant capacity by increasing NAD levels; however, this research is still in the early stages.
